# Comparative Genomics Analyses of Lifestyle Transitions at the Origin of an Invasive Fungal Pathogen in the Genus *Cryphonectria*

**DOI:** 10.1128/mSphere.00737-20

**Published:** 2020-10-14

**Authors:** Lea Stauber, Simone Prospero, Daniel Croll

**Affiliations:** a Swiss Federal Institute for Forest, Snow and Landscape Research (WSL), Birmensdorf, Switzerland; b Laboratory of Evolutionary Genetics, Institute of Biology, University of Neuchâtel, Neuchâtel, Switzerland; University of Georgia

**Keywords:** *Cryphonectria*, comparative genomics, lifestyle evolution, tree pathogen

## Abstract

Emerging fungal pathogens are a threat to forest and agroecosystems, as well as animal and human health. How pathogens evolve from nonpathogenic ancestors is still poorly understood, making the prediction of future outbreaks challenging. Most pathogens have evolved lifestyle adaptations, which were enabled by specific changes in the gene content of the species. Hence, understanding transitions in the functions encoded by genomes gives valuable insight into the evolution of pathogenicity. Here, we studied lifestyle evolution in the genus *Cryphonectria*, including the prominent invasive pathogen Cryphonectria parasitica, the causal agent of chestnut blight on *Castanea* species. We assembled and compared the genomes of pathogenic and putatively nonpathogenic *Cryphonectria* species, as well as sister group pathogens in the family Cryphonectriaceae (Diaporthales, Ascomycetes), to investigate the evolution of genome size and gene content. We found a striking loss of genes associated with carbohydrate metabolism (CAZymes) in *C. parasitica* compared to other Cryphonectriaceae. Despite substantial CAZyme gene loss, experimental data suggest that *C. parasitica* has retained wood colonization abilities shared with other *Cryphonectria* species. Putative effectors substantially varied in number, cysteine content, and protein length among species. In contrast, secondary metabolite gene clusters show a high degree of conservation within the genus. Overall, our results underpin the recent lifestyle transition of *C. parasitica* toward a more pathogenic lifestyle. Our findings suggest that a CAZyme loss may have promoted pathogenicity of *C. parasitica* on *Castanea* species. Analyzing gene complements underlying key nutrition modes can facilitate the detection of species with the potential to emerge as pathogens.

**IMPORTANCE** Forest and agroecosystems, as well as animal and human health, are threatened by emerging pathogens. Following decimation of chestnuts in the United States, the fungal pathogen *Cryphonectria parasitica* colonized Europe. After establishment, the pathogen population gave rise to a highly successful lineage that spread rapidly across the continent. Core to our understanding of what makes a successful pathogen is the genetic repertoire enabling the colonization and exploitation of host species. Here, we have assembled >100 genomes across two related genera to identify key genomic determinants leading to the emergence of chestnut blight. We found subtle yet highly specific changes in the transition from saprotrophy to latent pathogenicity mostly determined by enzymes involved in carbohydrate metabolism. Large-scale genomic analyses of genes underlying key nutrition modes can facilitate the detection of species with the potential to emerge as pathogens.

## Abstract (precis)

Forest and agroecosystems, as well as animal and human health, are threatened by emerging pathogens. Following decimation of chestnuts in the United States, the fungal pathogen *Cryphonectria parasitica* colonized Europe. After establishment, the pathogen population gave rise to a highly successful lineage that spread rapidly across the continent. Core to our understanding of what makes a successful pathogen is the genetic repertoire enabling the colonization and exploitation of host species. Here, we have assembled >100 genomes across two related genera to identify key genomic determinants leading to the emergence of chestnut blight. We found subtle yet highly specific changes in the transition from saprotrophy to latent pathogenicity mostly determined by enzymes involved in carbohydrate metabolism. Large-scale genomic analyses of genes underlying key nutrition modes can facilitate the detection of species with the potential to emerge as pathogens.

## INTRODUCTION

Across the fungal kingdom, species have evolved the ability to persist as either symbionts, commensals, or pathogens on a wide range of living insect, animal, and plant hosts. This variety of fungal lifestyles requires complex adaptations encoded in the genome. Lifestyle-associated adaptations have been of particular interest as pathogen emergence is frequently associated with a significant gain in virulence of a formerly weak pathogen (1). This has been shown for Pyrenophora tritici-repentis, a former saprophyte or weak pathogen on grass species including wheat, which became highly pathogenic on wheat through acquisition of the virulence gene *ToxA* from the wheat pathogen Stagonospora nodorum (2). Moreover, pathogen emergence can be promoted through host jumps or geographic range expansions (3) or complete host shifts (1). Such host shifts can occur across kingdoms, as shown for insect pathogens from the genus Metarhizium, which likely evolved from plant endophytes or pathogens (4). Interestingly, phylogenomic analyses have shown that pathogens can emerge repeatedly within fungal clades such as *Dothideomycetes* or even at the genus level (e.g., *Aspergillus*) (5, 6). Hence, many pathogenic fungi have nonpathogenic ancestors. This suggests that the emergence and evolution of pathogenic lifestyles are coupled with the acquisition of specific traits distinct from nonpathogenic relatives.

To be successful, pathogens must overcome physical and chemical barriers deployed by the host (7). Plant-pathogenic fungi have evolved specific lifestyles (i.e., biotrophy, hemibiotrophy, and necrotrophy) to exploit the host, and each lifestyle requires distinct sets of genes (8–11). The gene repertoire of pathogens evolved through gene gains or losses and proliferation of transposable elements, as well as expansions or contractions of entire gene families, sometimes resulting in increased genome sizes, compared to related nonpathogenic species (12, 13). Gene families notably associated with fungal plant pathogenicity include enzymes for cell wall degradation, small secreted proteins (i.e., effectors), and secondary metabolite gene clusters (14–19). Cell walls are an important physical barrier against pathogens but can be broken down and used as carbon sources by a variety of fungi. Carbohydrate-active enzymes (CAZymes) specific for cellulose, hemicellulose, or pectin degradation are typically classified into the superfamilies of glycoside hydrolases (GHs), glycosyl transferases (GTs), polysaccharide lyases (PLs), and carbohydrate esterases (CEs), as well as enzymes with auxiliary activities (AAs) and carbohydrate-binding modules (CBMs) (20). The types and number of CAZyme-encoding genes vary among species and likely reflect adaptation to different nutritional niches (21). Most notably, necrotrophic pathogens tend to deploy cell wall-degrading enzymes to promote host damage and colonization (22). In contrast, biotrophic pathogens tend to have fewer enzymes involved in cell wall degradation (20, 22). Saprotrophic fungi feeding on decaying plant matter often show an overall reduced CAZyme complement compared to necrotrophic fungi (23) but specific expansions in CAZymes related to cellulose degradation (24).

The emergence of pathogenic lifestyles has often required the ability to secrete effector proteins and secondary metabolites during contact with the host. Effectors are characterized as quickly evolving small, cysteine-rich secreted proteins, which are produced to manipulate plant host immune responses (25, 26). Biotrophic and hemibiotrophic pathogens secrete effector proteins to suppress host immunity and manipulate host cell physiology (27). Necrotrophs deploy effectors also as host-specific toxins (27, 28). However, small secreted proteins resembling effectors are also expressed by saprophytic fungi and may be involved in degradative processes (29). Virulence factors in pathogenic fungi can also include secondary metabolites, which are often low-molecular-weight compounds not essential for fungal growth. Polyketides, nonribosomal peptides, terpenes, and indole alkaloids are the main bioactive compounds acting as cytotoxins, antimicrobials, or enzyme inhibitors (30). Genes underlying secondary metabolite biosynthesis pathways are often clustered in the genome (31). Secondary metabolites are produced by fungi of various lifestyles but may be more relevant virulence factors for necrotrophs, while biotrophs tend to lose the underlying genes (8). Beyond pathogenicity-related functions, saprophytic or endophytic fungi produce secondary metabolites with important antimicrobial activity (32, 33).

The family Cryphonectriaceae (Diaporthales, Ascomycetes) includes mainly bark-inhabiting species ranging from weak to severe pathogens (34, 35). The most aggressive pathogens include *Chrysoporthe* species affecting hosts in the order Myrtales (e.g., *Eucalyptus* spp.), as well as Cryphonectria parasitica (Murr.) Barr., the causal agent of chestnut blight on *Castanea* (Fagaceae) species (36, 37). *C. parasitica* is native to East Asia (i.e., China, Korea, and Japan), where it occurs as a weak pathogen on Chinese (Castanea mollissima Blume) and Japanese (Castanea crenata Siebold & Zucc.) chestnuts. However, *C. parasitica* was first described after its discovery in 1904 on American chestnut [Castanea dentata (Marsh.) Borkh.] in the United States (37). The rapid spread of the pathogen following its introduction resulted in the ecological extinction of *Ca. dentata* throughout its native distribution range in North America (38). In Europe, chestnut blight was first observed in the 1930s and is nowadays present in all major chestnut-growing areas (37). Following the colonization of Europe, *C. parasitica* has rapidly spread through most of southeastern Europe, driven by the emergence of a highly successful lineage (39). The invasion success likely stems from the establishment of a highly diverse European bridgehead population and a switch to asexual reproduction (39). Besides host species in the genus *Castanea*, *C. parasitica* has been occasionally reported on oaks (*Quercus* spp.), maples (*Acer* spp.), and European hornbeam (Carpinus betulus L.) (37).

Both in the native and in the invasive range, *C. parasitica* has closely related sister species, which are considered weak pathogens or saprophytes (40). Among these, Cryphonectria japonica Tak. Kobay. & Kaz. Itô (previously named Cryphonectria nitschkei) was isolated from *Ca. crenata* in Japan (41, 42) and from oaks in China, on which it causes bark cankers (43). The European species Cryphonectria naterciae M.H. Bragança (syn. Cryphonectria decipiens [44]) was isolated from Castanea sativa and *Quercus* spp. in Portugal, Sardinia, and Algeria (45–47). Inoculation experiments showed that both *C. japonica* and *C. naterciae* are significantly less virulent on *Ca. sativa*, Quercus robur L., and Fagus sylvatica L. than *C. parasitica* (40, 43). Two other *Cryphonectria* species occurring in Europe are C. radicalis and C. carpinicola. The former is also present in North America and considered to be a saprophyte on dead wood of *Castanea* and *Quercus* species (48). Interestingly, the low prevalence may be the result of a displacement that occurred when the pathogenic sister species *C. parasitica* was first introduced to both continents (48). *C. carpinicola* is a recently described species isolated from declining European hornbeams in Austria, Georgia, Italy, and Switzerland (C. Cornejo, personal communication). The diversity of lifestyles within the Cryphonectriaceae, including the emergence of new pathogens, raises important questions of whether genetic factors facilitate pathogenic lifestyles.

In this study, we assembled and analyzed 104 genomes of the Cryphonectriaceae family including the major representatives *C. parasitica*, *C. radicalis*, *C. naterciae*, and *C. japonica* and a recently detected European *Cryphonectria* species named *C. carpinicola* (Cornejo, personal communication). We analyzed orthology among the gene sets of the species and constructed a robust phylogenomic tree. We find that Cryphonectriaceae share similar trophic lifestyle traits. However, the chestnut pathogen *C. parasitica* has a substantially reduced complement in CAZymes. In contrast, the capacity to produce secondary metabolites is reduced among *Cryphonectria* species but is broadly conserved within the genus. Effector candidate proteins show genus and species specificity consistent with faster evolvability of the underlying genes.

## RESULTS

### Genome assemblies for the *Cryphonectria* genus.

**FIG 1:**
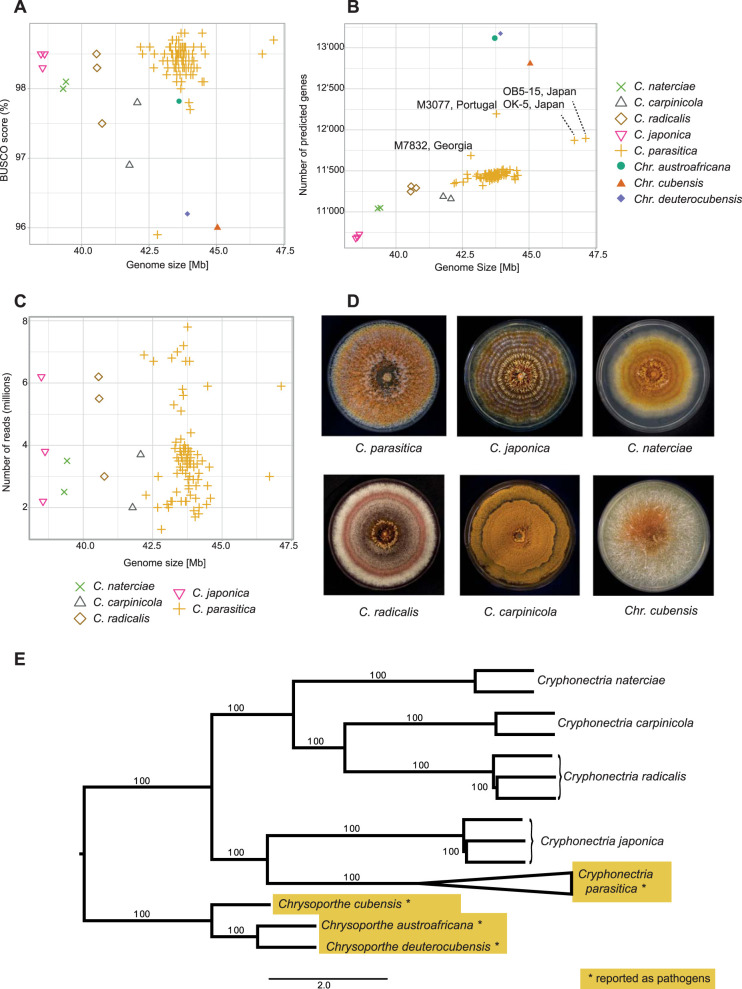
Assembly statistics and phylogenetic reconstruction. (A to C) Estimated genome size in megabases correlated with assembly completeness assessed by BUSCO scores (A), number of predicted genes (B), and sequencing depth of assembled Cryphonectriaceae genomes (C). (D) Culture morphology of the studied Cryphonectriaceae (cultures of *Chr. deuterocubensis* and *Chr. austroafricana* are not shown, as isolates were unavailable for documentation). (E) Maximum-likelihood consensus tree based on 6,770 single-copy ortholog genes showing the phylogenetic relationship of Cryphonectriaceae species.

We assembled draft genomes of 100 *Cryphonectria* species isolates of Asian, European, and North American origin, in addition to the previously assembled genome of *C. parasitica* reference genome EP155. As a near outgroup to the genus *Cryphonectria*, we analyzed previously assembled draft genomes of 3 *Chrysoporthe* species from South Africa, Colombia, and Indonesia. To assemble *Cryphonectria* genomes *de novo*, we used Illumina sequencing data at 9 to 53× coverage (Table 1). All *Cryphonectria* and *Chrysoporthe* genome assemblies showed >95% completeness for BUSCO genes (ascomycota_odb9 database) with the *C. parasitica* isolate M7832 having the lowest score at 95.9% (Table 1 and Fig. 1A). Based on the assembly size, we estimated that nonpathogenic species had smaller genomes ranging from 38.6 Mb (*C. japonica*) to 41.9 Mb (*C. carpinicola*). Pathogenic species had slightly larger genomes ranging from 43.7 Mb (*C. parasitica* and Chrysoporthe austroafricana) to 45 Mb (Chrysoporthe cubensis) (Table 1; Fig. 1A). We found no apparent correlation between the estimated genome size and the completeness in BUSCO genes (Fig. 1A and B). Similarly, we detected no correlation between the sequencing depth and the assembled genome size (Fig. 1C). This shows that the short-read-based assemblies are expected to reliably represent the gene content across species.

**TABLE 1:** Genome assembly statistics for *Cryphonectria* spp. and *Chrysoporthe* spp.^*a*^

Species	Mean size (Mb)	Mean *N*_50_	Mean complete BUSCO (%)	Coverage (min–max)	No. of predicted genes (min–max)	No. of isolates
C. parasitica*	43.7	125,044	98.40	9–53×	11,321–12,195	91
*C. japonica*	38.6	364,390	98.43	17–48×	10,680–10,729	3
*C. radicalis*	40.6	197,843	98.1	22–46×	11,247–11,312	3
*C. naterciae*	39.4	120,703	98.05	19–27×	11,041–11,050	2
*C. carpinicola*	41.9	58,390	97.35	14–26×	11,159–11,187	2
Chr. austroafricana*	43.7	48,708	97.8	NA	13,125	1
Chr. cubensis*	45.0	345,702	96	NA	12,807	1
Chr. deuterocubensis*	43.9	83,661	96.2	NA	13,174	1

a The *C. parasitica* reference genome is not included in the summary of *C. parasitica* genomes. Asterisks indicate pathogenic species. NA, not available.

### Gene annotation and phylogenetic reconstruction.

We predicted between ∼10,700 and 12,200 genes in genomes of *Cryphonectria* species compared to ∼12,800 to 13,170 genes in *Chrysoporthe* spp. (Table 1 and Fig. 1B). Overall, gene content among species was correlated with genome size except for *C. carpinicola* and *Chr. cubensis*, which have fewer predicted genes as expected from their genome size (Fig. 1B). Among *C. parasitica* isolates, M3077 had a higher gene content than isolates of similar genome size (Fig. 1B). Moreover, assembled genomes of *C. parasitica* isolates OB5-15 and OK-7 showed increased genome sizes while having only slightly higher gene content than other *C. parasitica* isolates (Fig. 1B).

The gene ortholog analyses revealed 6,770 single-copy orthologs among all species. We found 85 species-specific orthologs, of which 22 were specific for *C. parasitica*. Additionally, we found between 1 and 10 isolate-specific orthologs among the *C. parasitica* isolates TA51, M7832, DU5, OB5-15, OK-17, and M4030. Moreover, one ortholog was specific for *C. carpinicola*, while no species-specific orthologs were detected in all other *Cryphonectria* species. Within *Chrysoporthe*, we found 19 orthologs specific to Chysoporthe deuterocubensis, as well as 12 and 5 orthologs specific to *Chr. cubensis* and *Chr. austroafricana*, respectively. To reconstruct the evolutionary history of *Cryphonectria* and *Chrysoporthe* species, we generated a consensus maximum-likelihood tree based on 6,770 single-copy ortholog genes. We found 100% bootstrap branch support between species and a clear divergence at the genus level (Fig. 1E). Furthermore, *Cryphonectria* species were grouping by geographic origin, with *C. naterciae*, *C. radicalis*, and *C. carpinicola* being of European origin and *C. japonica* and *C. parasitica* being of Asian descent. Overall, our consensus tree is in accordance with phylogenetic studies on the genera *Cryphonectria* and *Chrysoporthe* (49; C. Cornejo, personal communication).

### Lifestyle prediction and capacity for carbohydrate metabolism across species.

**FIG 2:**
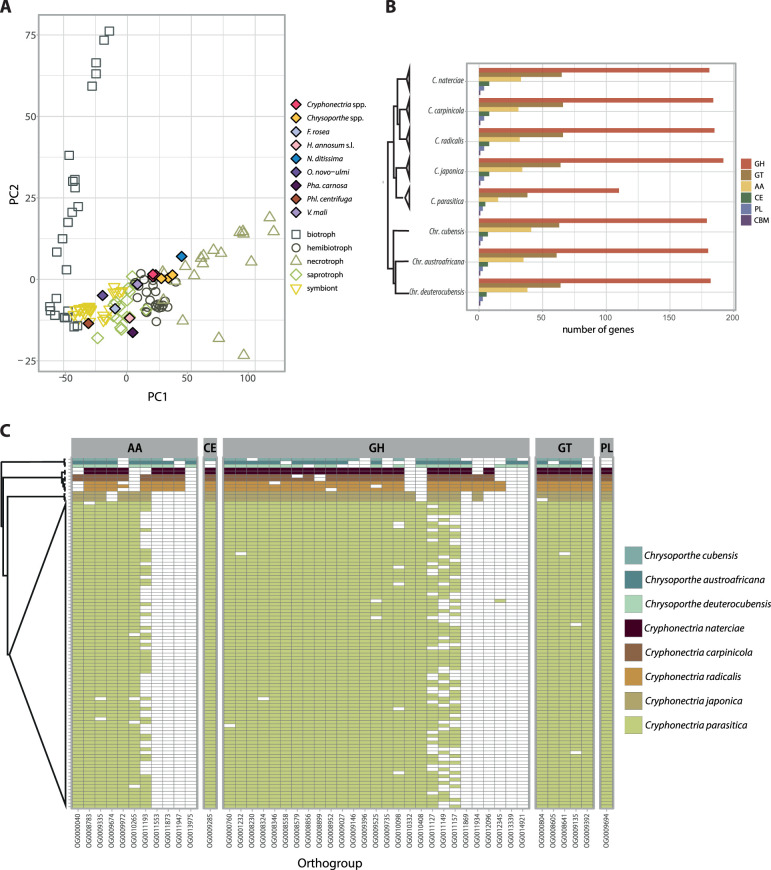
Carbohydrate-active enzyme (CAZyme) content among *Cryphonectriaceae*. (A) Principal-component analysis (PCA) of fungal lifestyle predictions, as inferred by CATAStrophy. The plot incorporates 85 reference species of fungi with different lifestyles (i.e., biotroph, hemibiotroph, nectrotroph, saprotroph, and symbiont) used as a training set by CATAStrophy and shows the CAZyme-inferred phenotypic trophism of Cryphonectriaceae and other pathogenic and nonpathogenic tree-associated fungi. (B) Number of detected CAZyme genes per species grouped according to CAZyme superfamily: glycoside hydrolase (GH), glycosyl transferase (GT), auxiliary activity (AA), carbohydrate esterase (CE), polysaccharide lyase activity (PL), and carbohydrate-binding modules (CBM). (C) Ortholog presence/absence of CAZyme superfamilies for which at least one species is missing an ortholog (the CBM superfamily is not shown, as orthologs were found in all species).

In order to degrade plant cell walls for nutrition or infection, fungi produce a variety of enzymes involved in carbohydrate metabolism (CAZymes) (50). We analyzed the predicted proteome of Cryphonectriaceae species and other tree-associated fungi to assess trophic lifestyles according to CAZyme content. All *Cryphonectria* species were identified as hemibiotrophs by CATAStrophy, while *Chrysoporthe* species were classified as necrotrophs. However, the principal-component analysis (PCA) shows close proximity of analyzed *Cryphonectria* and *Chrysoporthe* species, clustering at the verge with other hemibiotrophic and necrotrophic species (Fig. 2A). Lifestyles of most fungi outside the Cryphonectriaceae family matched with predicted lifestyles according to CAZyme content, except for Valsa mali (Fig. 2A).

We further assessed CAZyme gene content among Cryphonectriaceae and found a striking gene loss in the chestnut blight pathogen *C. parasitica* (Fig. 2B). The gene loss particularly affected the group of glycoside hydrolases (GHs), glycosyl transferases, and enzymes with auxiliary activity (AA). Overall, all nonpathogenic *Cryphonectria* species, as well as the pathogenic *Chrysoporthe* species, encoded between 38.5 and 42.7% more GH, 37.7 and 42.4% more GT, and 51.6 and 63.4% more AA than *C. parasitica* (Fig. 2B). We identified gene losses in *C. parasitica* across most CAZyme categories. GH5 associated with hemicellulose degradation showed a particularly remarkable reduction (see Fig. S2 in the supplemental material). We found between 12 and 13 GH5 genes in saprophytic *Cryphonectria* and 11 GH5 genes in *Chrysoporthe* spp., while *C. parasitica* had only four GH5 genes. Moreover, slightly fewer GH28 genes involved in pectin degradation were detected in *C. parasitica* (*n *= 11) than in *Chrysoporthe* spp. (*n *= 12 to 14) and saprophytic *Cryphonectria* (*n *= 15 to 16). Analyzing CAZymes for which at least one species is missing an ortholog, *Cryphonectria* species share a relatively conserved set of ortholog CAZyme genes as expected from their short phylogenetic distance (Fig. 2C). We found one PL orthogroup encoding pectate lyase (OG0009694), shared only among *Cryphonectria* species. Moreover, we detected one GH orthogroup belonging to the sialidase superfamily (OG0010332), which is present only in Asian *Cryphonectria* species, as well as a single GH orthogroup (OG0012096, GH3) present only in European *Cryphonectria* species (Fig. 2C). *C. parasitica* displayed a particularly high degree of intraspecific presence/absence variation for four auxiliary activity (AA) and GH enzymes, which are otherwise well conserved (OG0011193, GMC [glucose-methanol-choline] oxidoreductase; OG0011127, GH76; OG0011149, GH43; OG0011157, GH76) (Fig. 2C). The four orthogroups likely underwent recent gene losses in *C. parasitica.*

To assess the wood-colonizing capabilities of different *Cryphonectria* species and a member of the genus *Chrysoporthe* (*Chr. cubensis*), we conducted an inoculation experiment on dormant and healthy chestnut stems. We performed the experiment with and without prior removal of the bark. None of the species were able to colonize dormant chestnut logs without artificial wound induction. After 2 weeks of incubation, *C. japonica* showed signs of mycelial growth on the bark at a maximum of 1 cm beyond the inoculation point. No bark penetration was detected. For inoculations with bark removal, *C. parasitica* expectedly showed the fastest and most extensive lesion growth. Other *Cryphonectria* species, with the exception of one *C. radicalis* isolate (M4733), developed only minimal lesions (Fig. S1). We found intraspecific variance in lesion growth, possibly attributed to varying isolate vigor (e.g., *C. radicalis* isolate M283 was isolated in 1953) or variable substrate conditions (e.g., state of dormancy and stem thickness) (Fig. S1). The eucalyptus pathogen *Chr. cubensis* showed growth on nonhost chestnut (*Ca. sativa*) logs; however, lesions developed at a comparatively slow pace (Fig. S1). After 4 weeks of incubation, mycelial fans were found only in lesions caused by *C. parasitica*.

10.1128/mSphere.00737-20.1 FIG S1 Lesion length on dormant chestnut stems (*Castanea sativa*) with bark removal. Measurements were taken once per week during four weeks. Download FIG S1, PDF file, 1.2 MB. Copyright © 2020 Stauber et al. 2020 Stauber et al. This content is distributed under the terms of the Creative Commons Attribution 4.0 International license.

10.1128/mSphere.00737-20.2 FIG S2 Gene count of all identified CAZyme families among Cryphonectriaceae. Download FIG S2, PDF file, 0.2 MB. Copyright © 2020 Stauber et al. 2020 Stauber et al. This content is distributed under the terms of the Creative Commons Attribution 4.0 International license.

### Variation in secondary metabolite production potential among species.

**FIG 3:**
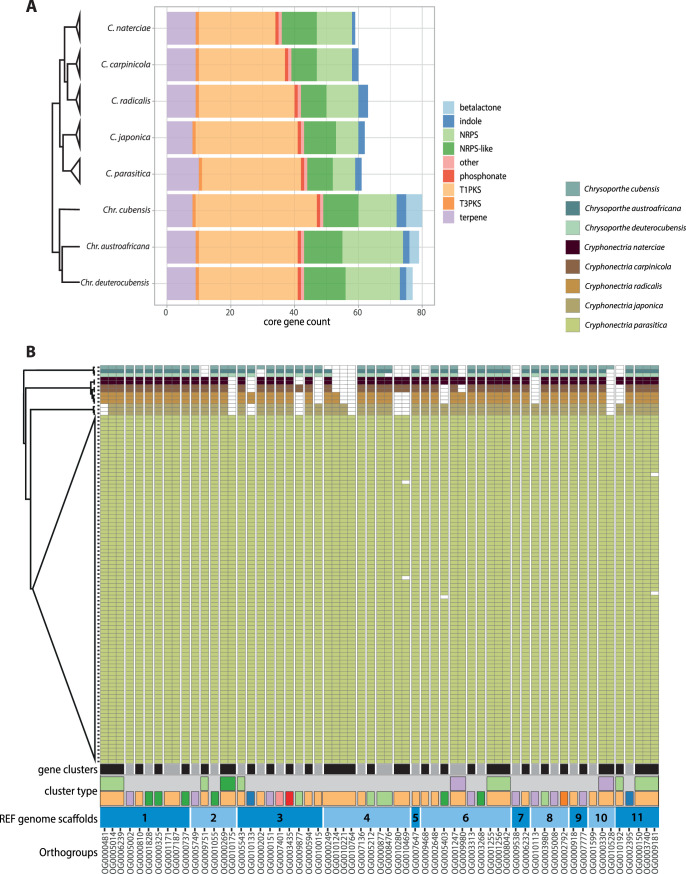
Secondary metabolite core gene content among *Cryphonectriaceae.* (A) Count of detected biosynthetic core gene categories across species as identified by antiSMASH. (B) Presence/absence of biosynthetic core gene orthologs among species. The plot shows the number of biosynthetic core genes within a gene cluster, the cluster type (color codes are as in panel A), and the location of clusters according to *C. parasitica* reference genome scaffolds.

Secondary metabolites (SMs) can play important roles in pathogenicity and the interaction with microbes (51, 52). We investigated variation in biosynthetic core genes as an indicator for metabolite production potential among species. Loss of a biosynthetic core gene from a cluster invariably leads to loss of cluster function. Overall, biosynthetic core gene counts were variable only between genera. *Cryphonectria* species had comparatively fewer biosynthetic core genes than *Chrysoporthe* species (Fig. 3A). Among the detected biosynthetic core genes, the two genera shared similar proportions of different gene cluster classes with type 1 polyketide synthase (T1PKS) being the most abundant gene cluster class (Fig. 3A). The class of beta-lactone production clusters, which can produce potent antibacterial and antifungal compounds (53), was exclusively found in *Chrysoporthe* species (Fig. 3A). The presence/absence analyses of biosynthetic core genes per gene cluster (*n *= 47) revealed 28 clusters conserved among all analyzed Cryphonectriaceae (Fig. 3B). Additionally, core genes in five clusters were conserved in *Cryphonectria*. The same clusters showed a partial or complete absence in *Chrysoporthe*. The largest cluster was found on scaffold 4, containing four T1PKS biosynthetic core genes. All four core orthologs of the cluster were retained in *C. parasitica.* Other species lost between one (*C. japonica*) and all four (*Chr. cubensis*) core genes (Fig. 3B). Overall, core genes were highly conserved among *C. parasitica* isolates, except for three T1PKS, nonribosomal peptide synthase (NRPS)-like, and NRPS-T1PKS clusters on scaffolds 4, 6, and 11 (OG0010469, OG0005403, and OG0009181) (Fig. 3B). The clusters showed gene losses in *C. parasitica* isolates from China (TA51), Georgia (M7776 and M7832), Japan (WB-3), and the United States (MD-1). Generally, we identified only weak homology with secondary metabolite clusters in other species. A notable exception includes two gene clusters potentially underlying emodin production (Fig. S3).

10.1128/mSphere.00737-20.3 FIG S3 Two PKS gene clusters (biosynthetic core gene orthologs OG0009468 and OG0002648) potentially underlying emodin production. Synteny plots of emodin and identified homologous gene clusters among species. Heatmaps show similarity (%) of genes in the *C. parasitica* reference genome cluster to emodin and orthologs in other Cryphonectriaceae (gray = gene is absent). Download FIG S3, PDF file, 0.4 MB. Copyright © 2020 Stauber et al. 2020 Stauber et al. This content is distributed under the terms of the Creative Commons Attribution 4.0 International license.

### Predicted effector genes among Cryphonectriaceae, effector orthologs, and cysteine content.

**FIG 4:**
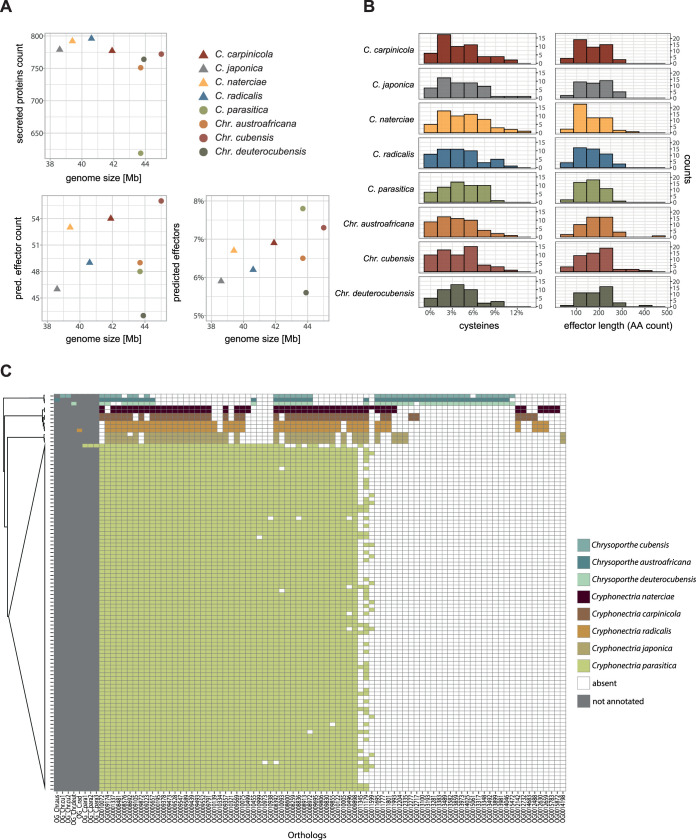
Predicted secretome and putative effectors among *Cryphonectriaceae*. Conserved orthologs (i.e., effector genes shared among all species) were omitted. (A) Genome size correlations with secreted proteins and predicted effectors (identified by EffectorP). Saprophytic species are shown with triangles, and pathogens are shown with circles. (B) Histograms showing the cysteine content (%) and the size of predicted effectors per species. (C) Presence/absence of predicted effector orthologs among species. Areas in gray show orthologs for which we found no corresponding ortholog.

Effectors are mostly secreted, cysteine-rich proteins, which play a major role in fungal virulence to overcome host immune defenses (9). We predicted effector genes with a machine-learning approach and found that neither the number of putative secreted proteins nor the predicted effector content correlated with genome size (Fig. 4A). Saprophytic *Cryphonectria* species encode slightly more putatively secreted proteins (*n *= 777 to 796) than pathogenic *Chrysoporthe* spp. (*n *= 751 to 772). Surprisingly, *C. parasitica* encodes markedly fewer secreted proteins (*n *= 619) than all other species (Fig. 4A). However, despite the small amount of secreted proteins, *C. parasitica* had the highest ratio of predicted effectors among all species with 7.8% of all secreted proteins predicted to function as effectors (Fig. 4A). Overall, the pathogenic versus saprophytic lifestyle did not correlate with predicted effector content. For example, we found that pathogenic *Chr. deuterocubensis* encoded the smallest number of predicted effectors of all analyzed species (Fig. 4A). The cysteine content of predicted Cryphonectriaceae effectors ranged from 0 to 12.9% (Fig. 4B). The predicted effectors among *Cryphonectria* contained 53 to 348 amino acids with one outlier of only 33 amino acids in *C. radicalis.* Predicted *Chrysoporthe* effectors contained 67 to 436 amino acids (Fig. 4B). The divergence in candidate effector gene content among Cryphonectriaceae matches the divergence in cysteine content and protein length. Analysis of predicted effector ortholog presence/absence among Cryphonectriaceae revealed 41.5% (*n *= 59) conserved orthologs in all Cryphonectriaceae, and 91 orthologs showed presence/absence variation among species (Fig. 4C). We found several orthologs unique to a single species (Fig. 4C). Interestingly, the species-specific *C. parasitica* orthologs OG0010999, OG0010973, and OG0010938 showed presence-absence variation with orthologs missing in isolates from China and South Korea (LB86, M8510, and S35) (Fig. 4C). For eight candidate effectors, we could not find a corresponding ortholog annotation with OrthoFinder (gray area in Fig. 4C).

## DISCUSSION

We assembled and analyzed genomes of eight bark-inhabiting Cryphonectriaceae species to retrace the evolution of genome size and gene content. Based on CAZyme content, all analyzed species are predicted to share a similar trophic lifestyle. In the genus *Cryphonectria*, we detected striking CAZyme gene loss in the invasive pathogen *C. parasitica*. In spite of the substantial CAZyme gene loss, *C. parasitica* shares wood colonization strategies with the other *Cryphonectria* species and has retained the ability for early saprotrophic wood decay. In contrast, secondary metabolite gene clusters diverged at the genus level but were largely conserved among *Cryphonectria* species. Putative effector content varied substantially among species with differences in cysteine content and protein length.

### Distinct CAZyme gene loss in a pathogenic species.

The CAZyme profiles of the Cryphonectriaceae species analyzed in this study match those of other hemibiotrophic or necrotrophic fungi. Thus, despite substantial difference in pathogenicity (40), Cryphonectriaceae species seem to share trophic lifestyle traits, which challenges previous classifications of *C. japonica*, *C. naterciae*, and *C. radicalis* as predominantly saprotrophic species (37). Nonetheless, the distinct CAZyme loss in *C. parasitica* coincides with an increased pathogenicity toward nonnative (i.e., non-Asian) *Castanea* species, which seems to be absent in other *Cryphonectria* species. Many CAZymes play a role in plant cell wall degradation and can be important virulence factors in necrotrophic fungi. Reductions in CAZyme genes have been observed in biotrophic pathogens and are thought to be an adaptation to reduce the exposure of molecular patterns, which can trigger host defenses (54, 55). Moreover, CAZyme loss can occur during host shifts, such as from plant to animal or insect hosts (56). In *C. parasitica*, the CAZyme loss may be an adaptation facilitating an increased pathogenic lifestyle. At the intraspecific level, fewer CAZymes are expressed during pathogenic growth compared to saprotrophic wood decay in the conifer pathogen Heterobasidion annosum *sensu lato*, which has plastic lifestyles (57). Similarly, *C. parasitica* may have undergone a transitory phase in the evolution of the predominant pathogenic lifestyle favoring reduced CAZyme expression and ultimately gene losses. Moreover, *H. annosum sensu lato* produces more secondary metabolites including phytotoxins during the pathogenic lifestyle (57). These findings suggest that necrotrophic pathogens of trees have evolved different wood degradation strategies from those of saprotrophic relatives. The most significant gene loss in *C. parasitica* was found in the CAZyme subfamily GH5, which underlies hemicellulose degradation. Consistent with this, GH5 expression is lower during pathogenic growth in *H. annosum sensu lato* (57). In contrast, genomes of saprotrophic wood degraders such as Phanerochaete carnosa have expanded GH5 repertoires (58). Despite extensive CAZyme loss in *C. parasitica*, our experimental data show that all *Cryphonectria* species, including *C. parasitica*, have retained similar wood colonization capabilities through bark wounds. Moreover, *C. parasitica* appears to have retained CAZymes suitable for early wood decay. This confirms field observations indicating that the fungus is able to survive a few years on the bark of fresh dead chestnut wood (59). In parallel to GH5, pectin-degrading enzymes of GH28 are also slightly reduced in *C. parasitica.* However, polygalacturonases belonging to the GH28 family are suggested to contribute to virulence in *C. parasitica* (60). Similarly, in other necrotrophic pathogens GH28 is also associated with pathogenicity showing expansions in the GH28 family (61). Similar to GH5, *C. parasitica* may have lost GH28 enzymes triggering host defenses through molecular pattern recognition by the host (62).

### Potential virulence-associated traits in *C. parasitica*.

In contrast to the evolution of CAZymes, secondary metabolite production capabilities are largely conserved within the genus *Cryphonectria*. *C. parasitica* produces virulence-associated compounds including oxalic acid, tannases, laccases, and phytotoxins such as cryparin and diaporthin, but the genetic basis is only partially resolved (60). The diaporthin production pathway is encoded by a PKS gene cluster in Aspergillus oryzae (63). However, we identified no clearly orthologous cluster in *C. parasitica*. The conservation of gene clusters across *Cryphonectria* species suggests that secondary metabolites played no particular role in the evolution of pathogenicity by *C. parasitica*. However, many fungi can modulate metabolite production depending on environmental conditions (64). Hence, even if all *Cryphonectria* species share a core set of gene clusters, lifestyle transitions may induce differential expression depending on biotic or abiotic conditions. In addition to secondary metabolites, small secreted proteins (i.e., effectors) can play key roles in the emergence of new pathogens. We identified a broad pool of putative effector orthologs among Cryphonectriaceae. The size of the effector gene pool did not correlate with genome size or lifestyle as seen in other clades of plant pathogens (27, 65). Effector homologs in the orthogroups OG0010999, OG0010973, and OG0010938 are particularly interesting candidates because the genes both are unique to *C. parasitica* and show presence/absence variation within the species. The recent gene gains in the pathogen lineage and the presence/absence variation within the species could explain variation in pathogenicity between and within the species, respectively. Combining analyses of positive selection, gene expression, and targeted gene deletion assays of effector candidates in *C. parasitica* will be needed to elucidate the role of effectors in causing chestnut blight.

### Lifestyle and the role of hosts.

Cryphonectriaceae species represent a useful model to retrace how lifestyle transitions toward pathogenicity impact the evolution of gene content. On its native Asian hosts (*Ca. crenata* and *Ca. mollissima*), the chestnut blight fungus *C. parasitica* causes only mild symptoms, which has been attributed to host-pathogen coevolution (37). In contrast, on the naive American and European chestnut species (*Ca. dentata* and *Ca. sativa*), the pathogen causes lethal bark cankers (66). In the invasive range, *C. parasitica* might also be a weak pathogen on *Quercus* spp., *Acer* spp., or *Carpinus betulus* (37). This suggests that *C. parasitica* has the genetic repertoire of a broad-host-range pathogen and that chestnut species may be the least able to resist pathogen invasion. In diverse forest ecosystems, disease incidence is often negatively correlated with host species richness (the “dilution effect” [67 to 69]). Hence, growth on largely resistant hosts may be a bet-hedging strategy of *C. parasitica* to survive and spread in the absence of the primary host (70). The weak pathogenicity of other *Cryphonectria* species may be facilitated by environmental conditions, such as abiotic stress on the host or disturbance of the host microbiome (71). Subsequently, these *Cryphonectria* species may be considered latent pathogens similar to some endophytes (72–74). Latent pathogenicity has been observed in other Cryphonectriaceae. For example, Granados et al. (75) found that the *Eucalyptus* pathogen *Chr. cubensis* is an endophyte on Colombian Melastomataceae trees. Moreover, the pathogen *Chr. austroafricana* occurs as an endophyte in its native range but is pathogenic on nonnative *Eucalyptus* trees (76). Hence, host jumps likely facilitated the switch from endophytic to pathogenic lifestyle in both species (75, 76). *C. parasitica* has recently emerged as a major pathogen on non-Asian chestnut species. To what degree the extensive CAZyme loss increased the pathogenic potential prior to the emergence as an invasive pathogen remains to be investigated. Comparative genomics combined with gene function analyses provide a powerful approach to study lifestyle evolution and changes in the underlying genome architecture.

## MATERIALS AND METHODS

### Genome sequencing.

We sequenced whole genomes of 90 *C. parasitica*, 3 *C. japonica*, 3 *C. radicalis*, 2 *C. naterciae*, and 2 *C. carpinicola* isolates covering the global distribution range (see Table S1 in the supplemental material). All isolates were prepared for sequencing as described in reference 39. Sequencing was conducted using the Illumina HiSeq4000 and Illumina NovaSeq 6000 platforms (Illumina, San Diego, CA, USA) at the Functional Genomics Center Zurich (FGCZ). By choosing the Illumina NovaSeq SP flow cell, NovaSeq reads were compatible with HiSeq4000 reads for downstream analysis.

10.1128/mSphere.00737-20.4 TABLE S1 Overview of genomes assembled in the genus *Cryphonectria*. Country, region, and host identify the location and host species of collection. Download Table S1, XLS file, 0.05 MB. Copyright © 2020 Stauber et al. 2020 Stauber et al. This content is distributed under the terms of the Creative Commons Attribution 4.0 International license.

### Genome assembly and gene prediction.

All 100 *Cryphonectria* sequences were assembled with SPAdes v3.13.0 (77), using the –careful option and choosing the k-mers 21, 33, 45, 57, and 69 for the iterative assembly process. Genome sizes and assembly quality of *Cryphonectria de novo* assemblies, as well as *Chrysoporthe* draft genomes, were assessed with QUAST v5.0.2 and BUSCO v3.0.2 (78, 79). Gene models were predicted using BRAKER2 v2.1.4 (80–83). Briefly, we set up gene annotation training using the existing *C. parasitica* v2 reference genome annotation (available from http://jgi.doe.gov/ [84]) using the BRAKER2 options –alternatives-from-evidence=false, –fungus, –gff3, and –skip_fixing_broken_genes. For splice site hints, intron information was extracted from the reference genome annotation using the construct_introns function from the R package gread v0.99.3 (85). After the training, genes were predicted in all assembled genomes using BRAKER2 adding coding sequence hints of the *C. parasitica* reference genome obtained using gffread v0.11.0 (86) and EMBOSS v6.6.0 tool transseq (87). We set the BRAKER 2 options –alternatives-from-evidence=false, –gff3, –useexisting, –prg=gth, and –trainFromGth.

### Identification of orthologs and secondary metabolite gene clusters.

To identify orthologs among all *Cryphonectria* and *Chrysoporthe* isolates, we used OrthoFinder v2.3.7 (88). We selected all single-copy ortholog groups and generated sequence alignments using MAFFT v7.429 (89). Aligned sequences were used for phylogenetic tree building by generating 100 maximum-likelihood (ML) trees using the GTRCAT model with RAxML v8.2.12 (90). The RAxML-generated tree and bootstrap files were subsequently used to build a consensus tree with Astral v5.14.2 (91). The obtained consensus tree was visualized with FigTree v1.4.3 (92). We used the antiSMASH fungal version v5.1.0 (93) to identify secondary metabolite gene clusters using one isolate per species: the reference genome EP155 for *C. parasitica*, IF-6 for *C. japonica*, M283 for *C. radicalis*, M3664 for *C. naterciae*, CS3 for *C*. *carpinicola*, and the three NCBI *Chrysoporthe* draft genomes. We used a custom Python script to extract the biosynthetic core genes from the antiSMASH regions.js file. The number of core genes per species was then plotted in R with the packages tidyverse (94), reshape2 (95), and ggplot2 (96). Additionally, we identified biosynthetic core genes per cluster of the EP155 genome and searched for orthologs in all species. Secondary metabolite core gene ortholog presence/absence in each cluster was plotted in R, using the packages reshape2, stringr (97), and ggplot2.

### Classification of fungal lifestyles according to CAZyme content.

We inferred trophic lifestyles of Cryphonectriaceae according to carbohydrate-active enzyme (CAZyme) gene content using the CAZyme-Assisted Training And Sorting of -trophy (CATAStrophy) prediction tool (23). CATAStrophy annotates CAZymes with HMMER 3.0 (98) and dbCAN (99) and predicts trophic classes based on a multivariate analysis (23). To run CATAStrophy, we selected the same Cryphonectriaceae isolates as described above and added additional tree-associated fungi of different lifestyles. For nonpathogenic saprophytes associated with wood degradation, we selected the proteomes of Fomitopsis rosea (BioProject accession no. PRJNA518053), Phanerochaete carnosa (58), and Phlebia centrifuga (100). Moreover, we included Heterobasidion annosum *sensu lato* (57), associated with both saprotrophic and pathogenic lifestyles, and the bark pathogens Neonectria ditissima (Nectria canker on apple and pear trees) (101), Ophiostoma novo-ulmi (Dutch elm disease) (102, 103), and Valsa mali (Valsa canker on apple trees) (104). For trophic lifestyle inferences, we used the CATASTrophy pipeline (https://github.com/ccdmb/catastrophy-pipeline), choosing the options -profile conda and –dbcan_version 8. The CATAStrophy literature-derived nomenclature (i.e., classification into biotrophs, hemibiotrophs, nectrotrophs, saprotrophs, and symbionts) was used for defining trophic lifestyles of species included in the CATAStrophy training set. Moreover, we selected principal components PC1 and PC2, which separate most training set species according to lifestyle for visualization (23).

### Analysis of carbohydrate-active enzyme genes (CAZymes) and inoculation experiments.

For the identification of CAZyme genes, we ran dbCAN v2.0.0 (99) on the same isolates as in the secondary metabolite analysis (i.e., one isolate per species). Only CAZymes which were identified by all three tools (HMMER, diamond, and hotpep) were then selected for further analysis. CAZyme orthologs were extracted using Python, and plots were generated in R.

To analyze the wood colonization capabilities of the different species, we set up an inoculation experiment. We selected 26 dormant chestnut logs (*Ca. sativa*; length, 50 cm; diameter, 3.3 to 6.7 cm), which were cut in a healthy state during winter from chestnut stands in Ticino, Switzerland, a week prior to the experiment. The logs were surface sterilized with 70% ethanol and sealed on both ends with paraffin to prevent desiccation. We selected 3 *C. parasitica* (XA19, CR03, and EP155), 2 *C. japonica* (M9249 and IF-6), 2 *C. naterciae* (M3664 and M3656), 2 *C. radicalis* (M4733 and M283), and 2 *C*. *carpinicola* (M9290 and CS3) isolates and 1 *Chr. cubensis* (CBS115724) isolate for inoculation. For all isolates except CBS115724, full genome sequences were available for this study. Prior to inoculation, all isolates were freshly inoculated from glycerol stocks onto potato dextrose agar (PDA; 39 ml/liter; BD Becton, Dickinson & Company, Franklin Lakes, NJ, USA) and incubated at 25°C in complete darkness for 5 days to induce mycelial growth. For inoculation of the first batch of chestnut logs (*n *= 13), we removed the bark on 5 equally distanced spots (diameter, 4 mm) on each log, placed a mycelial plug (diameter, 4 mm) into the wound, and sealed it with tape. For the second batch of chestnut logs (*n *= 13), we directly placed five mycelial plugs onto the bark of each log with equal distance (i.e., no wound induction) and sealed the inoculation spots with tape. For each treatment batch with and without wound, we selected 5 replicates per isolate and 5 negative controls (mycelium-free agar plugs), resulting in a total of 130 completely randomized inoculation spots (*n *= 65 per treatment). All chestnut logs were randomly placed onto racks in plastic containers, separated by treatment, filled with 2 liters of demineralized water to avoid drying out, and sealed with plastic lids (40). Incubation was at 20°C for both treatments. Logs with wounds were incubated for 4 weeks in complete darkness, and longitudinal lesion size was assessed once a week. Logs without wounds were incubated for 12 weeks, and lesion size was assessed at the end of the experiment.

### Prediction of effector genes.

We performed effector gene prediction with the same isolates as in the secondary metabolite and CAZyme analysis, using a machine-learning approach. First, secreted proteins were predicted with SignalP v5.0b (105), choosing the options -org euk and -format short. Only proteins with a likelihood probability >0.5 were selected for further analysis. Next, protein sequences with a predicted secretion signal were extracted with SAMtools v1.9 (106) and used as input for effector prediction with EffectorP v2.0 (107). Presence/absence variation analyses of predicted effector gene orthologs across species and plotting were performed in Python and R as described above. The cysteine content and protein length of predicted effector genes in all species were determined with EMBOSS pepstats v6.6.0.

### Data availability.

All sample accession numbers for the NCBI Short Read Archive, as well as NCBI accession numbers for all *de novo* assemblies of *Cryphonectria* genomes, are available in Table S1 in the supplemental material. Contigs of <400 bp were trimmed prior to NCBI genome submission. Outgroup genomes to the genus *Cryphonectria* were obtained for *Chrysoporthe cubensis*, *Chr. deuterocubensis*, and *Chr. austroafricana* from NCBI BioProjects PRJNA279968, PRJNA265023, and PRJNA263707, respectively (108, 109).
