# Supplementary figures and images for: Comparative Genomics Analyses of Lifestyle Transitions at the Origin of an Invasive Fungal Pathogen in the Genus Cryphonectria
Source: mSphere. 2020 Oct 14;5(5):e00737-20. doi: 10.1128/mSphere.00737-20 (PMC7565894; doi:10.1128/mSphere.00737-20)

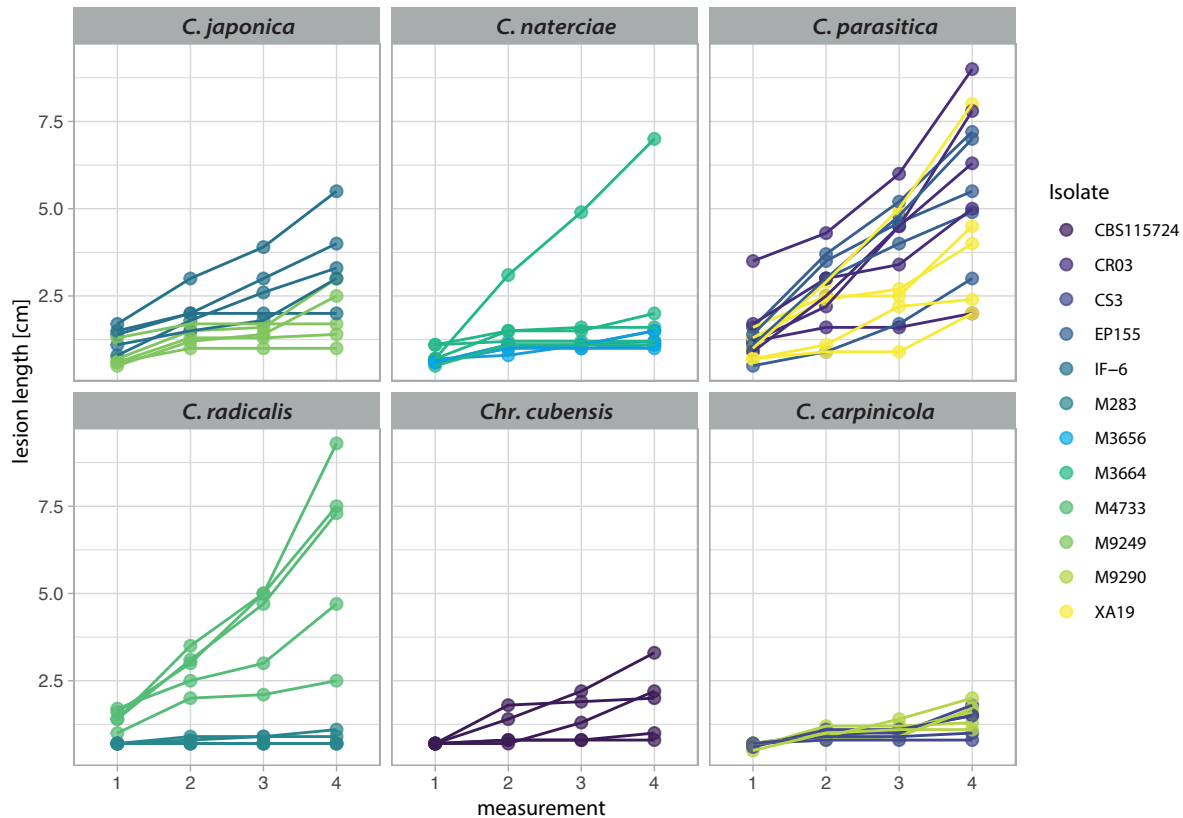

Supplement: FIG S1 [file mSphere.00737-20-sf001.pdf]

[illegible]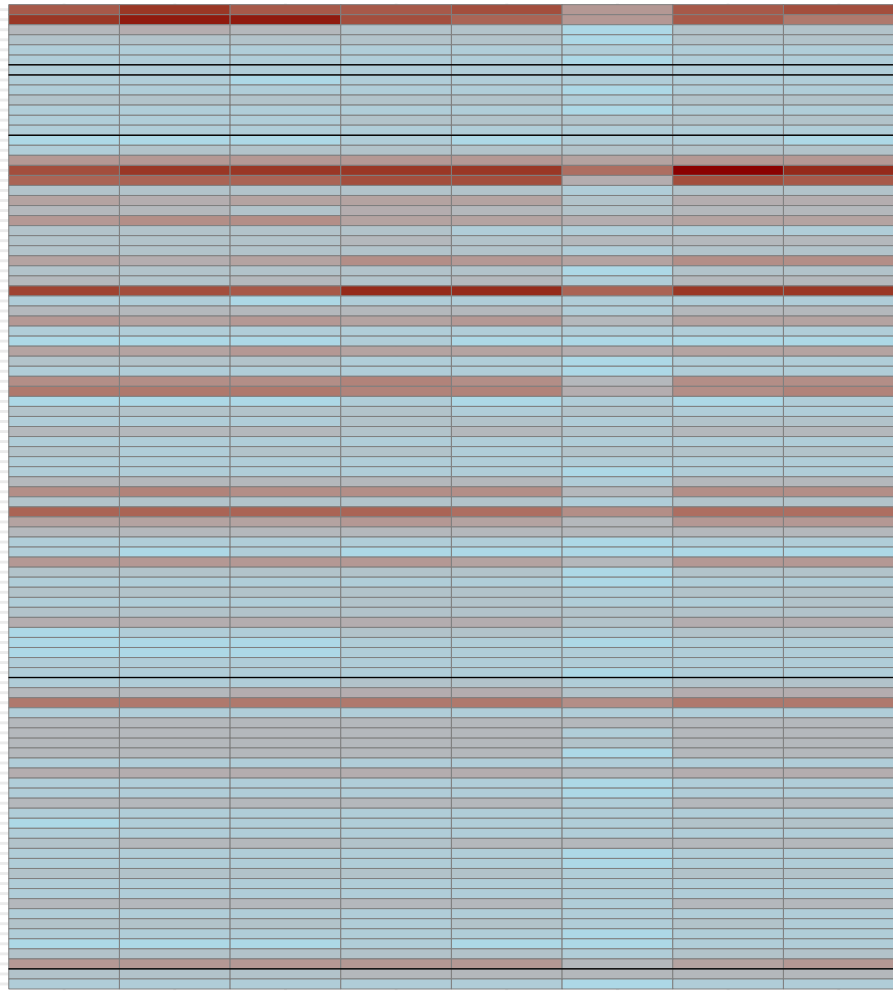

Supplement: FIG S2 [file mSphere.00737-20-sf002.pdf]

## OG0009468

*C. parasitica*  
VS

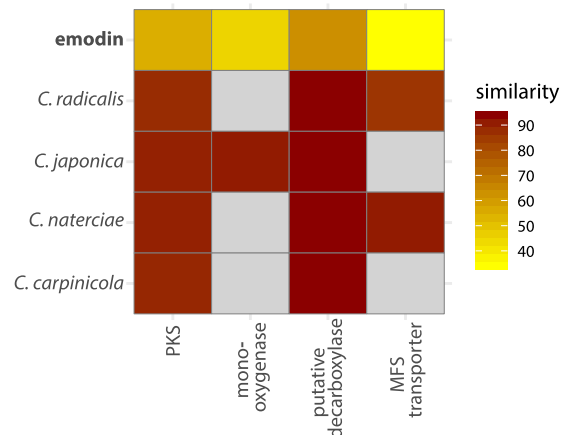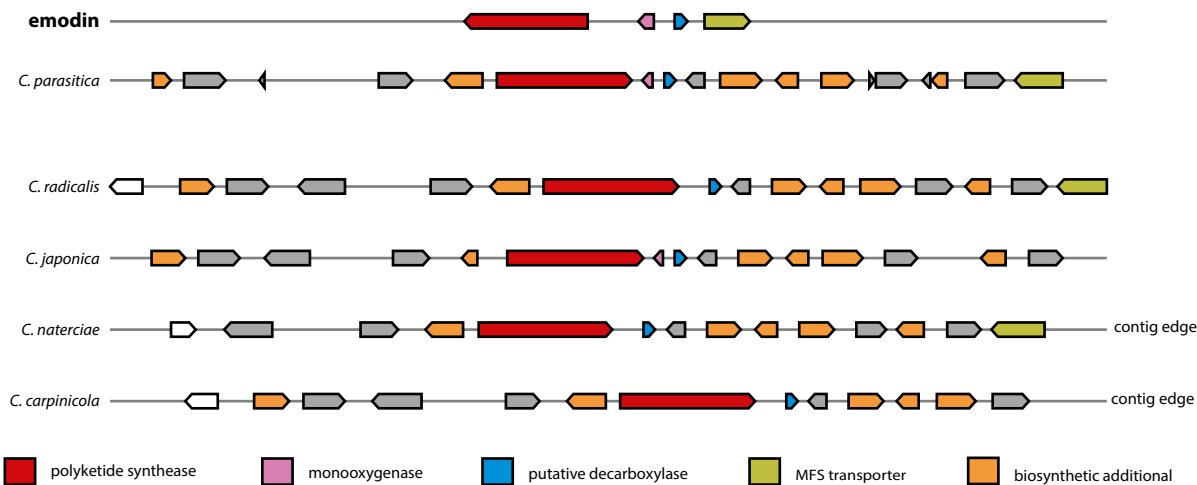

## OG0002648

*C. parasitica*  
VS

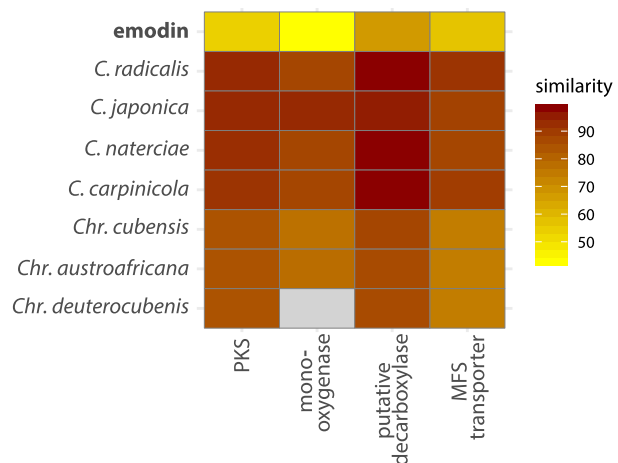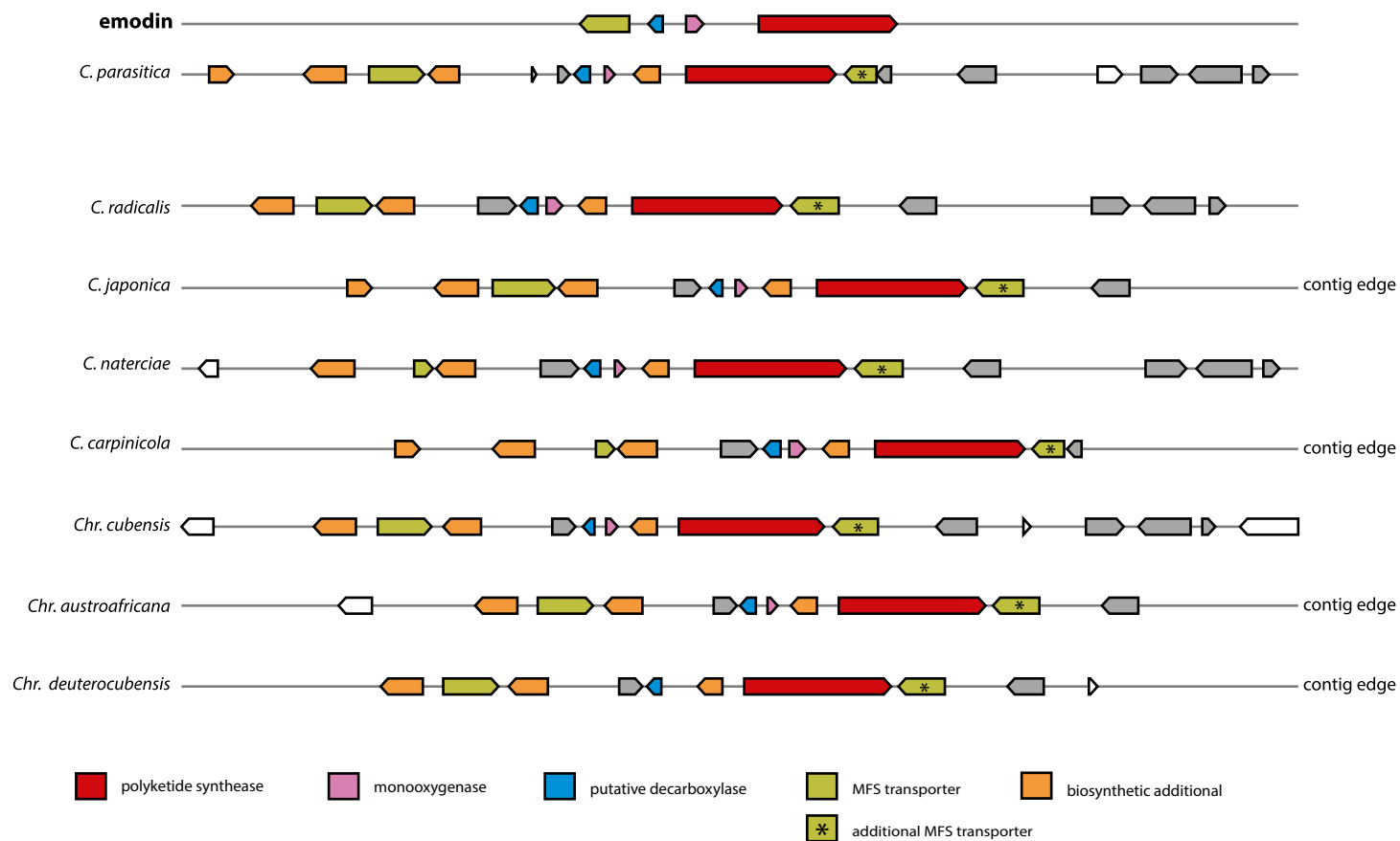

Supplement: FIG S3 [file mSphere.00737-20-sf003.pdf]
